# Contribution of Host Intracellular Transport Machineries to Intercellular Movement of Turnip Mosaic Virus

**DOI:** 10.1371/journal.ppat.1003683

**Published:** 2013-10-03

**Authors:** Maxime Agbeci, Romain Grangeon, Richard S. Nelson, Huanquan Zheng, Jean-François Laliberté

**Affiliations:** 1 INRS-Institut Armand-Frappier, Laval, Québec, Canada; 2 Plant Biology Division, Samuel Roberts Noble Foundation, Inc., Ardmore, Oklahoma, United States of America; 3 Department of Biology, McGill University, Montréal, Québec, Canada; IBMP CNRS Universit? de Strasbourg, France

## Abstract

The contribution of different host cell transport systems in the intercellular movement of turnip mosaic virus (TuMV) was investigated. To discriminate between primary infections and secondary infections associated with the virus intercellular movement, a gene cassette expressing GFP-HDEL was inserted adjacent to a TuMV infectious cassette expressing 6K_2_:mCherry, both within the T-DNA borders of the binary vector pCambia. In this system, both gene cassettes were delivered to the same cell by a single binary vector and primary infection foci emitted green and red fluorescence while secondarily infected cells emitted only red fluorescence. Intercellular movement was measured at 72 hours post infiltration and was estimated to proceed at an average rate of one cell being infected every three hours over an observation period of 17 hours. To determine if the secretory pathway were important for TuMV intercellular movement, chemical and protein inhibitors that blocked both early and late secretory pathways were used. Treatment with Brefeldin A or Concanamycin A or expression of ARF1 or RAB-E1d dominant negative mutants, all of which inhibit pre- or post-Golgi transport, reduced intercellular movement by the virus. These treatments, however, did not inhibit virus replication in primary infected cells. Pharmacological interference assays using Tyrphostin A23 or Wortmannin showed that endocytosis was not important for TuMV intercellular movement. Lack of co-localization by endocytosed FM4-64 and Ara7 (AtRabF2b) with TuMV-induced 6K_2_-tagged vesicles further supported this conclusion. Microfilament depolymerizing drugs and silencing expression of myosin XI-2 gene, but not myosin VIII genes, also inhibited TuMV intercellular movement. Expression of dominant negative myosin mutants confirmed the role played by myosin XI-2 as well as by myosin XI-K in TuMV intercellular movement. Using this dual gene cassette expression system and transport inhibitors, components of the secretory and actomyosin machinery were shown to be important for TuMV intercellular spread.

## Author Summary

Plant viruses move from the initially infected cell to neighboring cells during local movement and then over long distances through vascular tissue to establish a systemic infection in the plant. Virus intercellular transport requires viral and host factors to move viral RNA-protein complexes through plasmodesmata (PDs). Virus intercellular movement is normally assessed by assays that cannot always differentiate between reduced viral RNA replication and intercellular movement. By using a dual cassette of genes encoding fluorescent proteins that can differentiate between primary infected cells and cells infected after intercellular transport, we provide evidence that turnip mosaic virus (TuMV) needs a functional secretory pathway where pre- and post-Golgi trafficking and the actomyosin network are important for its movement. Interestingly, disruption of these host transport machineries had no impact on TuMV accumulation in initially infected cells. These results support the idea that virus replication activities can be influenced separately from those involved in other virus activities such as movement, although aspects of both are likely coordinated.

## Introduction

Plant viruses move from the initially infected cell to neighboring cells during local spread and then over long distances through vascular tissues to establish a systemic infection in the plant. Transport of viruses between cells first involves the intracellular movement of the viral RNA from the site of replication to plasmodesmata (PDs) and then its delivery into neighboring cells through PDs. PDs are tunnels in the cell wall that connect the cytoplasm, the endoplasmic reticulum (ER) and the plasma membrane between adjoining cells (reviewed in [1]). The size exclusion limit (SEL) of PD is normally too small to allow passive transport of large molecular complexes, but plant viruses encode movement proteins (MPs) that increase the SEL of PDs to allow passage of the viral RNA (reviewed in [2], [3]). Intracellular movement likely involves a membrane-associated viral RNA-host and viral protein complex, but the exact configuration of the viral entity that enters the neighboring cells has not yet been determined (reviewed in [2], [4]). In the case of tobacco mosaic virus (TMV), the viral RNA appears to spread between cells as membrane MP-associated viral replication complexes (VRCs) [5]. For members of the comovirus and caulimovirus genera, viral particles transit through MP-induced tubules that go through PDs for their delivery into non-infected cells [6]–[10].

Although MPs and other viral protein components are important for viral RNA intra- and intercellular movement, it is clear that host factors also are required for these activities. The cytoskeleton is an essential component of organelle trafficking in plant cells (reviewed in [11], [12]) and it has been shown to be involved in vertebrate virus intracellular movement (reviewed in [13]). In the case of TMV, several studies have shown that microtubules and microfilaments are necessary to anchor and release, or aid the movement of the VRC or MP granules often associated with ER (reviewed in [2], [4], [14]). Microfilaments influence the intracellular or intercellular transport of other MPs or viruses [15]–[20]. Myosin motors are also required for MP or viral trafficking [6], [21]–[24]. The secretory pathway is further involved in intra- and intercellular trafficking by several viruses [16], [17], [20], [21], [25], [26]. Finally, recent studies suggest that the endocytic transport pathway may be involved in viral movement [27], [28].

However, not all viruses or their components use the cytoskeleton or the secretory pathway for movement. For example, PD targeting of the tubule-forming MP of cowpea mosaic virus (CPMV) is not affected by either the disruption of ER-Golgi transport or by cytoskeleton disruption [29]. Similarly, the targeting of the triple gene block protein 3 (TGBp3) of poa semilatent virus to PD does not require a functional cytoskeleton or the secretory pathway for its intracellular transport [30].

The genome of potyviruses is a single ∼10 kb RNA molecule that codes for a polyprotein, which is processed into ten mature proteins. In addition to polyprotein-derived polypeptides, an ∼7 kDa protein termed PIPO is produced in infected cells [31] and is also found as a trans-frame protein consisting of the amino-terminal half of P3 fused to PIPO (P3N-PIPO) [32]. Potyviruses have no designated MP but many viral proteins have been reported to have MP-related functions. For instance, HCPro and the coat protein (CP) can increase PD SEL [33]. In addition, CP and cylindrical inclusion (CI) protein are required for virus intercellular movement [34]–[36] and are associated with PD [37], [38]. Recently, the targeting of CI to PD was shown to be mediated by P3N-PIPO [39], which itself is targeted to the plasma membrane through an interaction with the host protein PCaP1 [32]. One last protein involved in viral movement is the 6 kDa membrane-associated 6K_2_ protein. It induces the production of motile vesicles that contain viral RNA and have been proposed to be the vehicle for intracellular trafficking of potyviral RNA [40], [41]. These findings, while providing a partial understanding of the mechanism of TuMV intracellular and intercellular movement, do not sufficiently explain the involvement of the host secretory pathway or the cytoskeleton in potyvirus movement.

In this study, we used a novel dual gene cassette construct that differentiated primary infected cells from cells infected after virus intercellular movement to show that the early as well as the late secretory pathway, but not endocytosis, was important for TuMV transport. We also determined that myosin XI-2 and XI-K, but not myosin XI-F and VIIIs, influenced TuMV intercellular movement. Although these cellular components were required for intercellular movement of TuMV, they did not appear to be involved in the virus protein production in primary infected cells.

## Results

### An *in vivo* quantitative assay for TuMV intercellular movement

**Figure 1:**
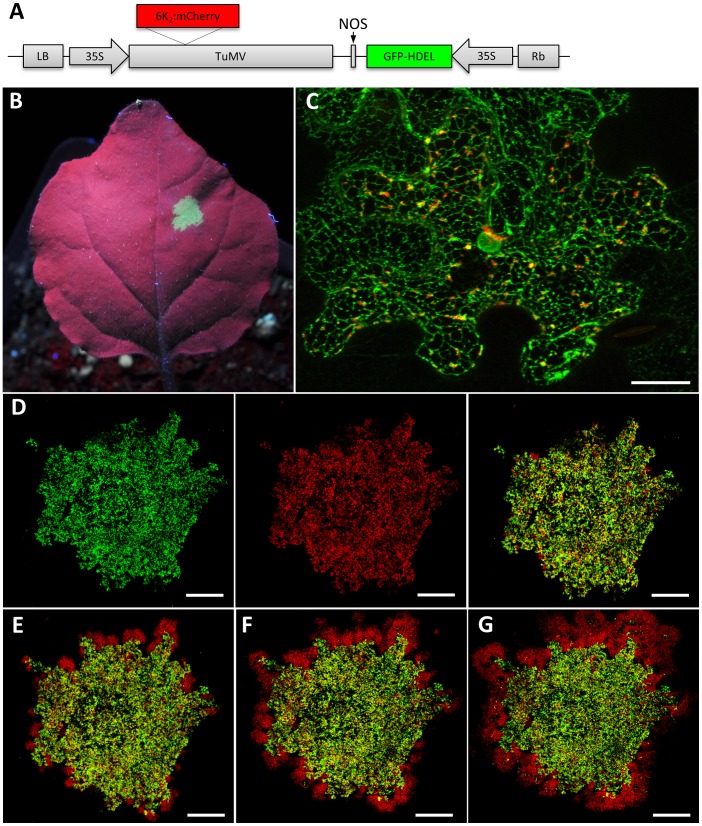
TuMV intercellular movement time course. (A) Schematic representation of the plasmid pCambiaTuMV/6K_2_:mCherry//GFP-HDEL used to discriminate primary infected cells from secondary infected cells after agroinfiltration. cDNA coding for 6K_2_:mCherry was inserted between P1 and HCPro cistrons. (B) Image of a leaf under uv illumination. Green fluorescing zone shows agroinfiltrated area. (C) Three-dimensional rendering of 35 1 µm thick confocal image sections that overlap by 0.5 µm showing the distribution of TuMV-induced 6K_2_:mCherry-tagged structures and GFP-HDEL labeled at 72 hpinf. The TuMV-induced 6K_2_:mCherry-tagged structures represent the viral factories, and the circular green structure in the center of the cell is the nucleus. Scale bar = 20 µm. (D) Three-dimensional Tile imaging rendering of *N. benthamiana* leaf agroinfiltrated 60 hrs before with *A. tumefaciens* strain Agl1 containing the above plasmid. The confocal image tiles was formed using the 10× objective by assembly 12×12 images in xy and the three-dimensional rendering was created by 5 z stacks of 90 µm thick confocal images that overlap by 45 µm. Left panel, red fluorescence channel imaging TuMV producing 6K_2_:mCherry; middle panel, green fluorescence channel imaging GFP-HDEL; and right panel, merged images. (E–G) Same infiltrated area as in D but confocal images were taken at 4, 5 and 6 dpinf. Scale bar = 2.5 mm.

A recombinant tobacco etch virus (TEV) (genus *Potyvirus*) engineered to express the reporter protein β-glucuronidase (GUS) allowed direct observation of virus spread in leaves [34]. Virus spread is influenced by the rates of virus RNA replication and virus intercellular movement. Hence, the use of the above TEV-GUS construct to fully interpret results from virus spread studies is limited since virus replication in live tissue cannot be quantitated through GUS staining. In order to discriminate initially infected cells from later infected cells in live tissue, we introduced within the T-DNA borders of a binary vector a gene cassette expressing the ER-localized GFP-HDEL adjacent to a TuMV infectious genome cassette expressing 6K_2_:mCherry (Fig. 1A). Since both gene cassettes are delivered to the same cells and GFP-HDEL does not move between cells [28], primary infected cells should display concomitant green and red fluorescence while secondary infected cells should display red-only fluorescence. This system also allows differentiation between virus RNA replication and virus intercellular movement.

A single infiltration with an *A. tumefaciens* suspension containing the above plasmid was performed on leaves of three-week old *N. benthamiana* plants, resulting in an agroinfiltrated area of 5–10 mm in diameter (Fig. 1B). Fluorescence emitted by GFP-HDEL was generally observed at approximately 36 hrs post infiltration (hpinf) and mCherry fluorescence resulting from TuMV replication was detected at approximately 60 hpinf. Systemic TuMV infection was observed at 4–5 days post infiltration (dpinf) in leaves above the infiltrated one by Western blot analysis using a rabbit serum against the CP of TuMV (data not shown). A similar systemic movement was obtained when more dilute agrobacterium suspensions (e.g. 0.01–0.001) were infiltrated, indicating the bacterial load did not elicit a plant defense response that might have affected virus infection rate. *N. benthamiana* cells displayed the expected green polygonal ER pattern and virus-induced 6K_2_-tagged red vesicles (Fig. 1C). Virus intercellular movement was assayed by observing red and green fluorescence at the perimeter of the infiltrated area. At 72 hpinf, a majority of cells in the infiltrated area emitted both green and red fluorescence and just a few red-only cells were observed (Fig. 1D), indicating that viral movement was just starting. Viral movement was followed in the same agroinfiltrated region at 4, 5, and 6 dpinf (Fig. 1E–G). At the end of the observation period, the surface area of green fluorescence did not changed, indicating that GFP-HDEL did not moved into neighboring cells.

Intercellular movement of fluorescent signal was also followed by observing spread of green and red fluorescence for 17 consecutive hours starting at 72 hpinf in order to evaluate the rate of cell-to-cell movement. This was achieved by securing an infiltrated leaf still attached to the plant on the confocal microscope stage for observation. When the perimeter of the agroinfiltrated area was initially imaged, intercellular movement have already begun as indicated by the presence of red-only fluorescent patches (Movie S1). To calculate the rate of cell-to-cell movement in cells/hour, we first measured the number of leaf epidermal cells for every linear 1 mm in three-week-old *N. benthamiana* plants and we calculated that 1 mm corresponded to 17.6 cells (n = 20). We then measured the distance from the agroinfiltrated front to the limit of expansion of the red fluorescence at the end of the observation period. In this experiment, the red-only fluorescence, indicative of TuMV secondary infection, had spread an average distance of 311 µm (n = 20) in the xy plane in 17 h. This expansion corresponded to a rate of one new infected cell every 3 hours. We repeated this experiment five times and we observed the same rate of cell-to-cell movement. This rate of intercellular movement was similar to that observed for TEV expressing GUS and for TMV [5], [34]. The increase in red fluorescence surface area was not due to the diffusion of 6K_2_:mCherry from agroinfected cells in the absence of virus spread because replacement of the TuMV cassette with a cassette expressing only 6K_2_-mCherry did not produce red-only fluorescent foci (Fig. S1A). These findings validated the use of the double cassette, GFP-HDEL and TuMV 6K_2_:mCherry to follow intercellular movement by TuMV.

### Intercellular movement of TuMV requires both the early and late secretory pathways

Chemical and protein inhibitors were used to evaluate the role of the early and late secretory pathways in the intercellular movement of TuMV using the dual gene cassette system. The plant secretory pathway consists of the ER, the Golgi apparatus, various post-Golgi intermediate compartments (e.g. trans-Golgi network, endosomes), the vacuoles/lysosomes and the small vesicular transport carriers that shuttle between these compartments. The early secretory pathway embraces the ER–Golgi interface while the Golgi apparatus and the various post-Golgi organelles that control plasma membrane or vacuolar sorting is categorized as the late secretory pathway (reviewed in [42]).

**Figure 2:**
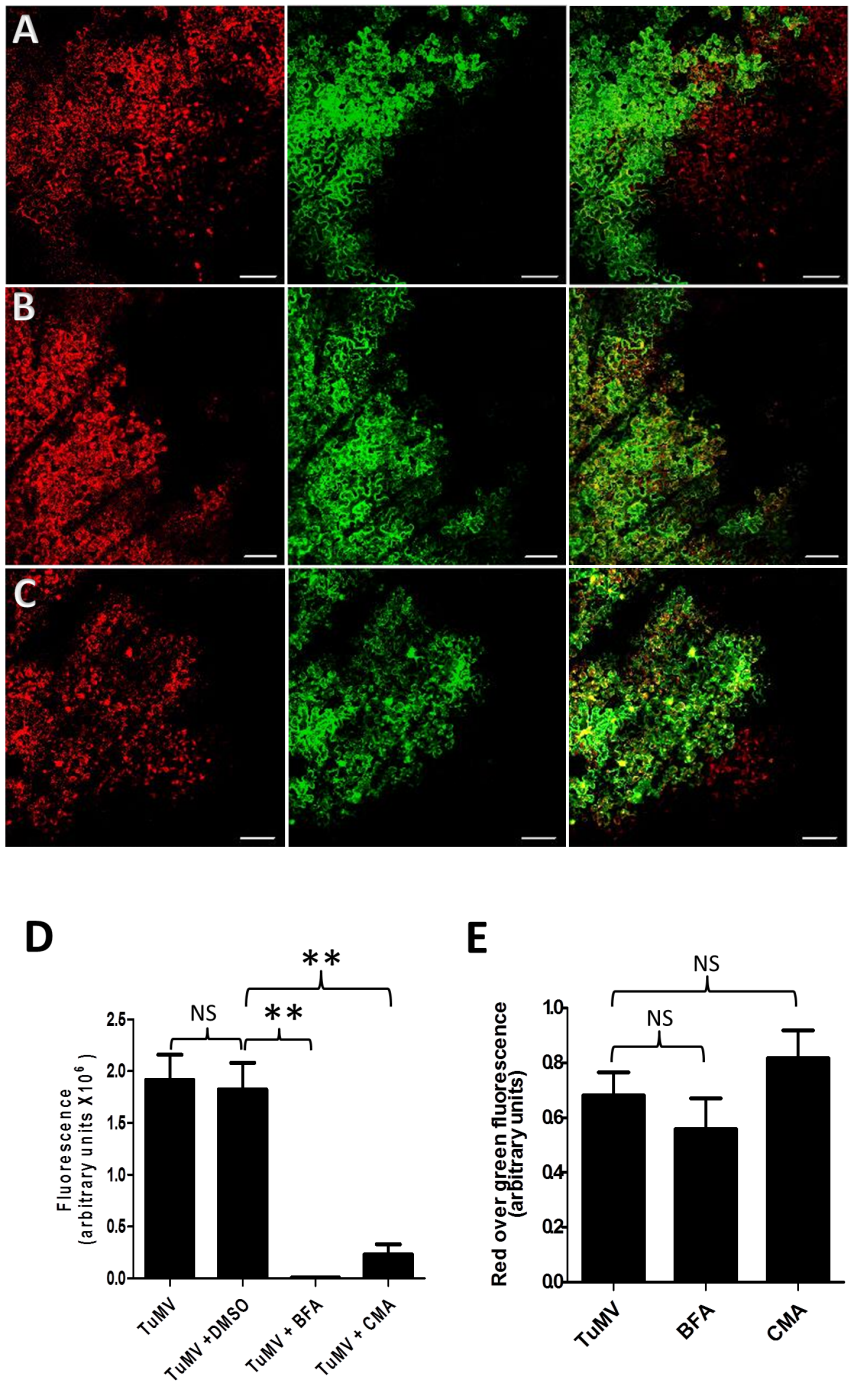
The secretory pathway is required for intercellular movement. *N. benthamiana* leaves were infiltrated with DMSO (A), 10 µg/ml BFA (B) and 0.5 µM CMA (C) 4 hours before agroinfiltration with *A. tumefaciens* containing pCambiaTuMV/6K_2_:mCherry//GFP-HDEL. All images were taken at 4 dpinf. Left panel, red fluorescence channel imaging TuMV producing 6K_2_:mCherry; middle panel, green fluorescence channel imaging GFP-HDEL; and right panel, merged images. Scale bar = 200 µm. (D) Surface area of red-only fluorescent foci was calculated and expressed in fluorescence units. (E) Fluorescence intensity ratio of red over green foci was calculated and expressed in fluorescence units. Bars represent means and standard errors for 20 replicates per treatment. One-way analysis of variance calculation followed by Tukey's Multiple Comparison Test allowed analysis of differences between means: **, 0.001<P value<0.01.

Brefeldin A (BFA) is an inhibitor that interferes with protein transport between the ER-Golgi interface [43]. Concanamycin A (CMA) inhibits protein transport at the trans-Golgi network (TGN) [44] by inhibiting the function of TGN-localized proton-ATPases, which leads to the acidification of the TGN lumen [45]. To evaluate the influence of these secretory inhibitors on TuMV intercellular movement, *N. benthamiana* leaves were treated with DMSO, 10 µg/mL BFA or 0.5 µM CMA 4 h before pCambiaTuMV/6K_2_:mCherry//GFP-HDEL agroinfiltration. BFA at this concentration effectively blocked the secretory pathway (Fig. S1B). At 4 dpinf, DMSO alone had no inhibitory effect on TuMV movement (Fig. 2A). On the other hand, BFA and CMA treatment reduced cell-to-cell movement of TuMV (Fig. 2B–C). The surface area for mCherry-only expressing foci for each treatment was measured and the statistical analysis confirmed the inhibitory effect of BFA and CMA on TuMV intercellular movement (Fig. 2D). This experiment was repeated two more times and the statistical data are presented in Fig. S2A. To assess whether or not BFA or CMA inhibited TuMV replication, we quantified mCherry fluorescence intensity over GFP fluorescence intensity in primary infection foci for all treatments. Fig. 2E shows that that there was no significant difference in the ratio of red over green fluorescence during BFA and CMA treatments compared with the no inhibitor treatment (TuMV alone) at 4 dpinf, indicating that viral protein production in the primary infected cells was not affected by the drug treatments. Green fluorescence levels were also similar between DMSO- and BFA- or CMA-treated primary cells indicating that steady state level of GFP, which was not associated with virus replication, was not affected by the treatments (data not shown).

**Figure 3:**
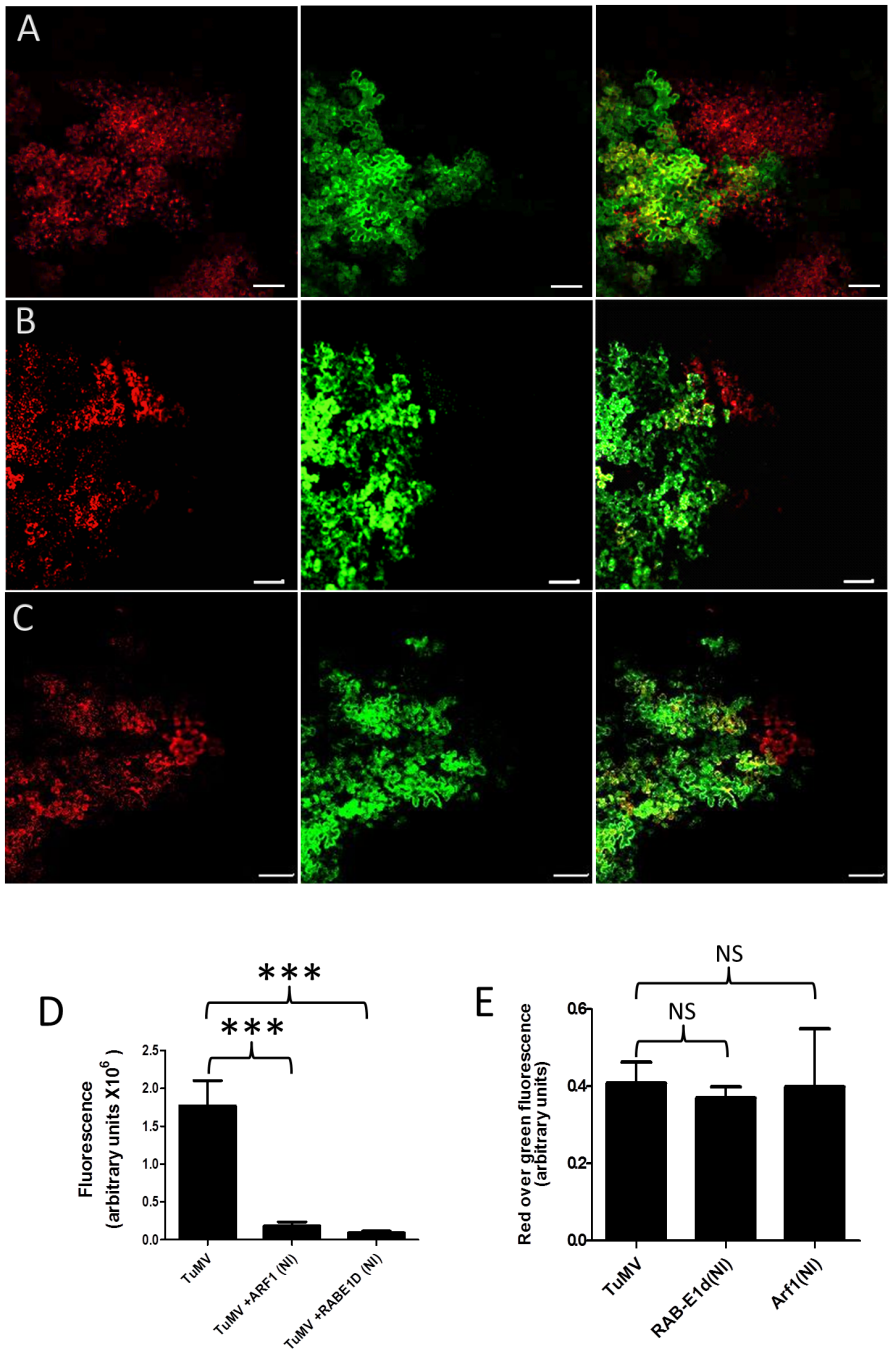
Inhibition of TuMV intercellular movement by dominant negative mutants of secretory pathway factors. *N. benthamiana* leaves were agroinfiltrated with *A. tumefaciens* containing plasmids pCambiaTuMV/6K_2_:mCherry//GFP-HDEL alone (A) or with dominant negative mutant ARF1(NI) (B) or with RAB-E1d (NI) (C). All images were taken at 4 dpinf. Left panel, red fluorescence channel imaging TuMV producing 6K_2_:mCherry; middle panel, green fluorescence channel imaging GFP-HDEL; and right panel, merged images. Scale bar = 200 µm. (D) Surface area of red-only fluorescent foci was calculated and expressed in fluorescence units. (E) Fluorescence intensity ratio of red over green foci was calculated and expressed in fluorescence units. Bars represent means and standard errors for 20 replicates per treatment. One-way analysis of variance calculation followed by Tukey's Multiple Comparison Test allowed analysis of differences between means: ***, P value<0.0001.

Protein inhibitors were used to further support the role of the secretory pathway in TuMV intercellular transport. The ADP-ribosylation factor 1 (ARF1) is a small GTPase regulating the recruitment of COPI coatomer proteins. A dominant negative mutant of ARF1 [ARF1(NI)] impaired in GTP/GDP binding inhibits the transport of soluble markers from the ER to Golgi, and causes a re-absorbance of Golgi membrane proteins into the ER [46]. RAB-E1d is a small Rab GTPase acting at a post-Golgi trafficking pathway and the dominant negative mutant RAB-E1d(NI) inhibits trafficking from the Golgi apparatus to the plasma membrane [47]. These two dominant-negative mutants were co-infiltrated with pCambiaTuMV/6K_2_:mCherry//GFP-HDEL. Four days post-agroinfiltration, red-only foci were reduced in the tissue co-infiltrated with the two mutant protein cassettes compared with those not infiltrated with these constructs, indicating reduced intercellular movement of TuMV in the presence of these secretory pathway inhibitors (Fig. 3B–C). Surface area measurements for mCherry-only expressing patches confirmed the inhibitory effect of both ARF1(NI) and RAB-E1d(NI) on TuMV intercellular movement (Fig. 3D). This experiment was repeated two more times and the statistical data are presented in Fig. S2B. Expression of these two mutant proteins did not hamper virus protein production in primary infected cells as measured by red over green fluorescence ratios in the dual expressing regions of the infected leaves (Fig. 3E). We therefore concluded that inhibition of both early and late secretory pathways inhibited TuMV intercellular movement but did not affect virus replication in primary infected cells. The last assertion is in line with the prior observation that BFA treatment did not affect the production of TuMV-induced 6K_2_-tagged perinuclear structures and peripheral vesicles [48].

### Intercellular movement of TuMV does not depend on the endocytic pathway

We examined if endocytosis was involved in TuMV intercellular movement. To this end, we first used a pharmacological interference assay with Tyrphostin A23, Tyrphostin A51 and Wortmannin. In mammalian cells, Tyrphostin A23 inhibits the recruitment of endocytic cargo into clathrin-coated vesicles formed at the plasma membrane by preventing the interaction between the clathrin-binding AP-2 adaptor complex μ2 subunit and the sorting motif within the cytoplasmic domain of plasma membrane proteins [49]. Tyrphostin A51 is a structural analog of Tyrphostin A23 but has no inhibitory effect and is routinely used as negative control [49]. Tyrphostin A23 is active in plant cells [50] and it has been shown that the drug inhibits endocytosis of some plasma membrane proteins [51]. Wortmannin is a phosphatidylinositol 3-kinase inhibitor that inhibits in mammalian cells receptor sorting and/or vesicle budding required for delivery of endocytosed material to “mixing” endosomes [52]. In plant cells, it has been shown that the drug inhibits endocytosis of FM4-64 (an amphiphilic styryl dye used to monitor endocytosis) [53] and morphogenesis of MVB/PVCs [50], [54] but it does not affect protein transport from the trans-Golgi network (TGN) to the plasma membrane [55], [56]. *N. benthamiana* leaves were infiltrated either with Tyrphostin A23, Tyrphostin A51, Wortmannin or DMSO 4 hrs prior to agroinfiltration with *A. tumefaciens* Agl1 containing pCambiaTuMV/6K2:mCherry//GFP-HDEL.

**Figure 4:**
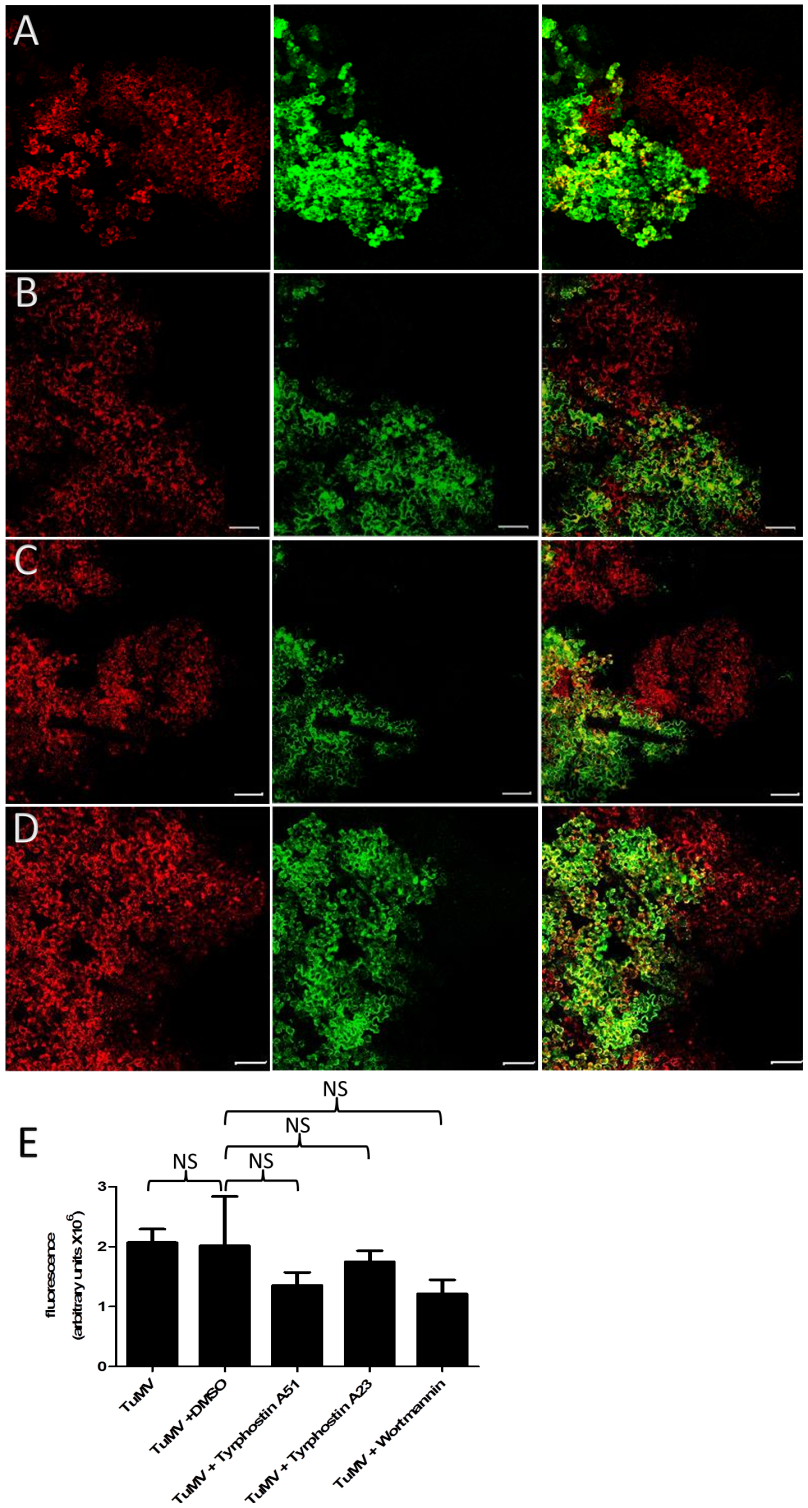
TuMV intercellular movement does not depend on the endocytic pathway. *N. benthamiana* leaves were infiltrated with DMSO (A), 20 µM Wortmannin (B), 30 µM Tyrphostin A51 (C) and 30 µM Tyrphostin A23 (D) 4 hours before agroinfiltration with *A. tumefaciens* containing pCambiaTuMV/6K_2_:mCherry//GFP-HDEL. Images were taken at 4 dpinf. Left panel, red fluorescence channel imaging TuMV producing 6K_2_:mCherry; middle panel, green fluorescence channel imaging GFP-HDEL; and right panel, merged images. Scale bar = 200 µm. (E) Surface area of red-only fluorescent foci was calculated and expressed in fluorescence units. Bars represent means and standard errors for 15 replicates per treatment. One-way analysis of variance calculation followed by Tukey's Multiple Comparison Test allowed analysis of differences between means: NS, not significant.

The drug concentrations used previously were shown to block endocytotic pathway in plants [50], [54] and inhibition of endocytosis of FM4-64 in the presence of Wortmannin was confirmed in our system (Fig. S1C). Four days post agroinfiltration, TuMV movement was examined (Fig. 4B–E). TuMV intercellular spread was not significantly inhibited in the presence of the different drugs that modify cellular endocytotic activity. This experiment was repeated two more times and data are presented in Fig. S2C. Thus maintenance of TuMV intercellular movement does not require endocytic activity within the four day period of observation.

**Figure 5:**
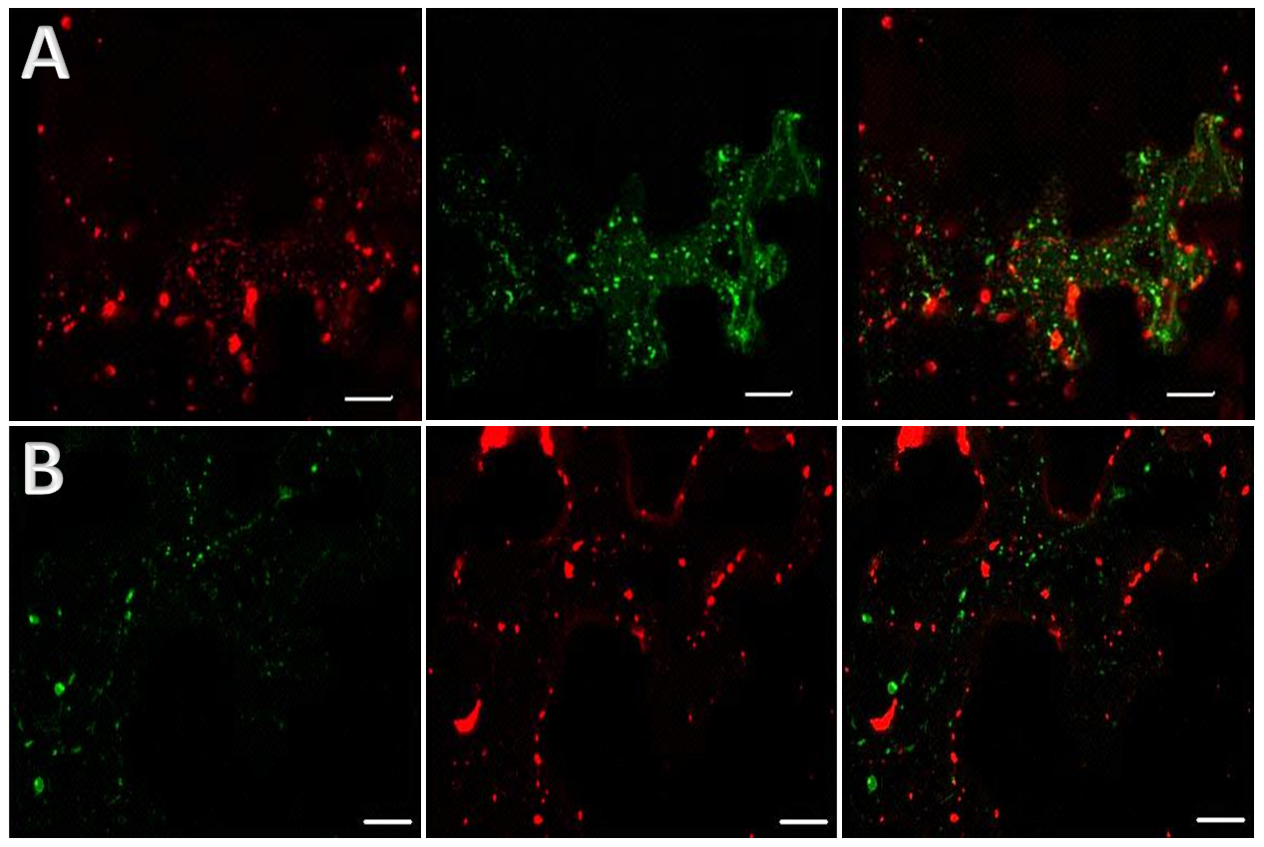
TuMV 6K_2_-tagged vesicles do not colocalize with endocytic markers. Three-dimensional rendering of 22 1 µm thick confocal images that overlap by 0.5 µm of *N. benthamiana* agroinfiltrated leaves. (A) shows the distribution of TuMV-induced 6K_2_:mCherry-tagged structures and YFP labeled Ara7 dots. Left panels; red fluorescence channel imaging TuMV producing 6K_2_:mCherry, middle panel; green fluorescence channel imaging YFP-RabF2b, and right panel; merged images. (B) shows the distribution of TuMV-induced 6K_2_:GFP-tagged structures and FM4-64 labeled vesicles. Left panel shows green fluorescence channel from TuMV producing 6K_2_:GFP, middle panel red fluorescence channel from FM4-64, and right panel merged images. Images were taken at 4 dpinf. Scale bar = 20 µm.

We also investigated whether TuMV-induced 6K_2_-tagged vesicles were associated with Ara7 (AtRAB-F2b) and internalized FM4-64. Intracellular trafficking of 6K_2_-tagged vesicles, which contain viral RNA [40], [41], has been shown to be dependent on the secretory pathway and microfilaments [40], [48], [57]. Ara7 is a plant Rab protein similar to Rab5 of mammals and to Ypt51/Ypt52/Ypt53 of yeast, and is associated with prevacuolar compartments and involved in endocytic and vacuolar trafficking in plant cells [58], [59]. Co-expression of Ara7 fused to GFP and pCambiaTuMV/6K_2_:mCherry in *N. benthamiana* leaves cells showed that there was no colocalization between Ara7 motile dots and 6K_2_-tagged vesicles (Fig. 5A). Also, 6K_2_-tagged vesicles were never associated with FM4-64-labeled vesicles (Fig. 5B). Lack of co-localization of FM4-64 and Ara7 with TuMV-induced 6K_2_-tagged vesicles further indicates that endocytic pathways associated with these markers were not important for TuMV cellular spread.

### Intercellular movement of TuMV depends on myosin XI motors

**Figure 6:**
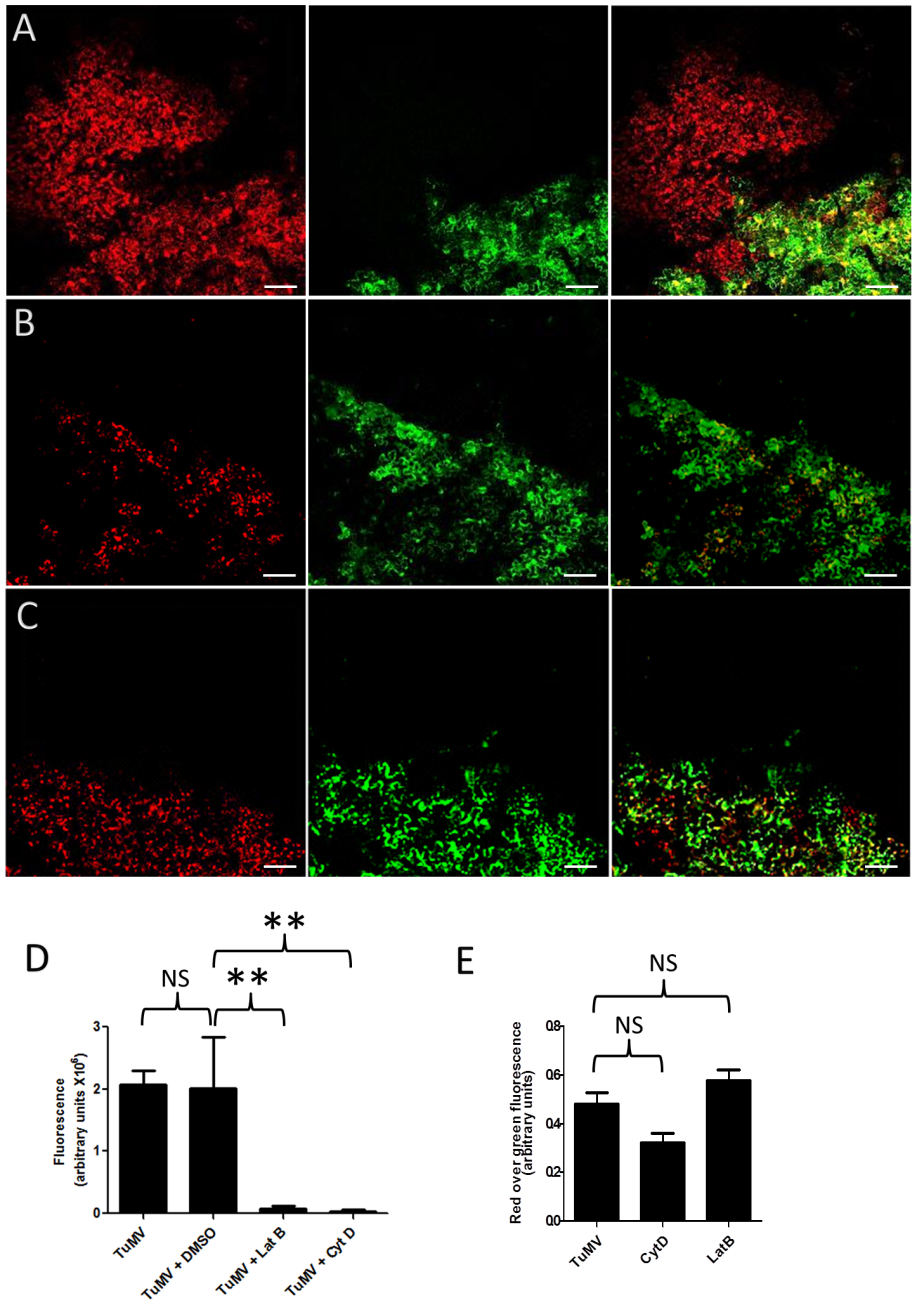
Microfilament network is required for TuMV intercellular movement. *N. benthamiana* leaves were infiltrated with DMSO (A), 5 µM LatB (B), and 10 µM CytD (C) 4 hours before agroinfiltration with *A. tumefaciens* containing pCambiaTuMV/6K_2_:mCherry//GFP-HDEL. Images were taken at 4 dpinf. Left panel, red fluorescence channel imaging TuMV producing 6K_2_:mCherry; middle panel, green fluorescence channel imaging GFP-HDEL; and right panel, merged images. Scale bar = 200 µm. (D) Surface area of red-only fluorescent foci was calculated and expressed in fluorescence units. (E) Fluorescence intensity ratio of red over green foci was calculated and expressed in fluorescence units. Bars represent means and standard errors for 20 replicates per treatment. One-way analysis of variance calculation followed by Tukey's Multiple Comparison Test allowed analysis of differences between means: NS, not significant, **, 0.001<P value<0.01.

Many viruses and individual virus proteins require the actomyosin system for their intracellular and/or intercellular movement [15], [16], [18]–[20], [22], [60]. However, recent studies showed that RNA viruses might have evolved differently in their requirements for actin and the associated myosin motors [22], [61]. We first tested the effect of Latrunculin B (LatB), and Cytochalasin D (CytD), which inhibit maintenance of microfilaments [62], on the intercellular spread of TuMV. Leaves were infiltrated with 5 µM LatB, 10 µM CytD, or DMSO 4 h before agroinfiltration with pCambiaTuMV/6K_2_:mCherry//GFP-HDEL. The disruption of actin by LatB or CytD was confirmed by confocal microscopy observation of microfilaments labeled with the actin-binding domain 2 of *A. thaliana* fimbrin fused to GFP (GFP-ABD2-GFP) [63] (Fig. S1D). TuMV intercellular movement was assessed by imaging *N. benthamiana* leaves at 4 dpinf. Inhibition of TuMV intercellular movement was observed after LatB or CytD treatment (Fig. 6B–D). This experiment was repeated two more times and the statistical data are presented in Fig. S3A. The ratio of red to green fluorescence in dual expressing cells was unchanged between treatments indicating that virus replication was unaffected by these microfilament antagonists (Fig. 6E). These results indicate that an intact microfilament network was important for TuMV intercellular movement, but not for replication. The last assertion is in line with the prior observation that LatB treatment did not affect the production of TuMV-induced 6K_2_-tagged perinuclear structures and peripheral vesicles [40].

**Figure 7:**
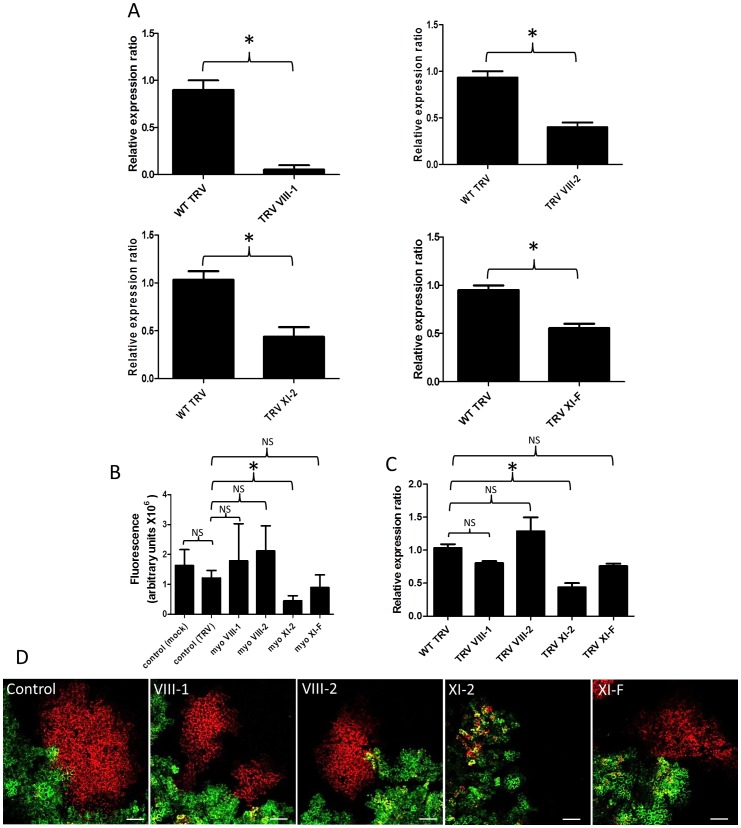
Myosin XI-2 is implicated in TuMV intercellular movement. (A) Quantitative RT-PCR was used to determine the relative expression ratio of target genes (myosin VIII-1, myosin VIII-2, myosin XI-2, and myosin XI-F) in *N. benthamiana* infected with the indicated TRV silencing constructs versus a TRV control not expressing a myosin fragment. (B) *N. benthamiana* leaves silenced for individual myosin genes (myosin VIII-1, myosinVIII-2, myosin XI-2, and myosin XI-F) were agroinfiltrated with pCambiaTuMV/6K_2_:mCherry//GFP-HDEL and surface area of red-only fluorescent foci was calculated and expressed in fluorescence units at 4 dpinf. Wild-type TRV (TRV) or buffer (Mock) were used as controls. Bars represent means and standard errors for 10 replicates per treatment. One-way analysis of variance calculation followed by Tukey's Multiple Comparison Test allowed analysis of differences between means: NS, not significant; *, P<0.05. (C) Level of expression of non-target myosins in *N. benthamiana* leaves silenced for myosin XI-2. The internal loading control for each sample was actin-2. Expression analysis was performed on extracts from systemic leaves at 20 dpinf with TRV constructs. Bars represent means and standard errors for three replicates per treatment. One-way analysis of variance calculation followed by Tukey's Multiple Comparison Test allowed analysis of differences between means: = NS, not significant; *, P<0.05. The experiment was repeated twice for each TRV silencing construct. (D) images of pCambiaTuMV/6K_2_:mCherry//GFP-HDEL in *N. benthamiana* leaves silenced for individual myosin genes (VIII1, VIII-2, XI-2, XI-F). Scale bar = 200 µm.

It was previously shown that overexpression of the myosin XI-K tail, a dominant negative mutant of this myosin species, inhibited the intracellular trafficking of TuMV 6K_2_ vesicles and reduced TuMV infection [41], indicating the involvement of this myosin in virus movement. We were also interested to see if other myosins could be involved. Tobacco rattle virus-mediated virus-induced silencing (TRV-VIGS) was adopted to determine the role of myosins on intercellular movement of TMV, potato virus X (PVX), tomato bushy stunt virus, and turnip vein-clearing virus (TVCV) [22]. We thus used TRV-VIGS to silence individual myosin genes prior to TuMV infection. *N. benthamiana* leaves were first infected with TRV constructs and 15 days later upper leaves were infiltrated with agrobacterium strain containing pCambiaTuMV/6K_2_:mCherry//GFP-HDEL. TuMV intercellular movement was quantified at 4 dpinf. Quantitative RT-PCR confirmed that the transcriptional level of the target myosin genes was decreased in plants infected by the TRV silencing construct containing the corresponding genes (Fig. 7A). We then monitored TuMV intercellular movement by measuring areas of foci expressing mCherry-only. Quantification indicated that there was no significant difference in TuMV intercellular movement in mock- and TRV-infected plants (Fig. 7B and 7D). Virus movement was not affected in myosin VIII-1 and VIII-2-silenced plants. However, silencing of myosin XI-2 reduced TuMV intercellular movement by a factor of 10 compared to the control (Fig. 7B and 7D). Reduced TuMV movement was observed in myosin XI-F silenced plants, but was not found to be statistically significant. This experiment was repeated two more times and the data are presented in Fig. S3B. To be sure this effect on virus movement was specific to myosin XI-2 silencing, we analyzed the effect of silencing myosin XI-2 on the other myosins (Fig. 7C). Silencing myosin XI-2 had no significant effect on the transcript level of the other tested myosins.

**Figure 8:**
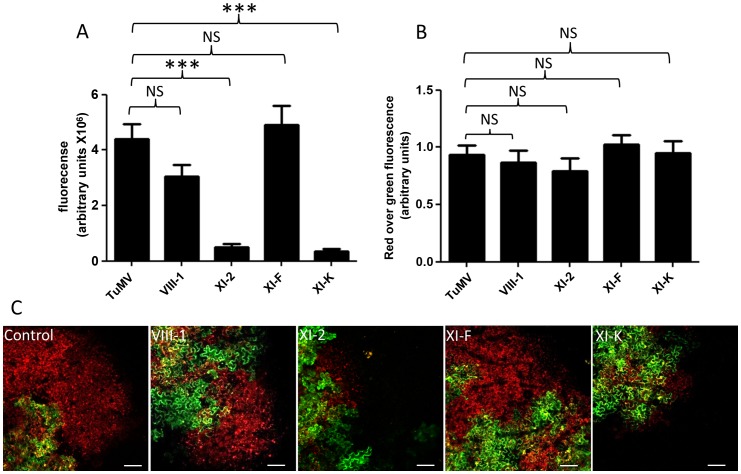
Myosin XI-2 and XI-K are implicated in TuMV intercellular movement. *N. benthamiana* leaves were agroinfiltrated with dominant negative myosin mutants and 24 h later with pCambiaTuMV/6K_2_:mCherry//GFP-HDEL. (A) Surface area of red-only fluorescent was calculated and expressed in fluorescence units at 4 dpinf. Bars represent means and standard errors for 21 replicates per treatment. (B) Fluorescence intensity ratio of red over green foci was calculated and expressed in fluorescence units. One-way analysis of variance calculation followed by Tukey's Multiple Comparison Test allowed analysis of differences between means: = NS, not significant, ***, 0.0001<P value<0.001. (C) Images of pCambiaTuMV/6K_2_:mCherry//GFP-HDEL in *N. benthamiana* leaves expressing dominant-negative myosin mutants (VIII1, XI-2, XI-F and XI-K). Scale bar = 200 µm.

Studying the role of different myosins in virus movement has also been carried out with transient expression of dominant negative myosin mutants [6], [23]. A significant decrease of movement was observed when we expressed *N. benthamiana* dominant negative myosin mutants for the myosin XI-2 and XI-K, but no significant effect was observed for myosin VIII-1 and XI-F (Fig. 8A and C). This experiment was repeated two more times and the data are presented in Fig. S3C. Intensity ratio of red over green foci was calculated to determine if replication was affected by the expression of the dominant-negative myosin mutants. The replication was not affected by any dominant-negative myosin mutants assessed in this experiment (Fig. 8B). Results presented here indicate that myosin XI-2 and XI-K are required for intercellular movement of TuMV and that the other myosins analyzed did not appear to play a role in cell-to-cell movement of TuMV.

## Discussion

Studies on intercellular movement have shown that plant viruses may use different trafficking pathways to move from one cell to another (reviewed in [2], [4]). In this study, by discriminating infiltrated and primary-infected cells from cells infected following intercellular virus movement, we were able to evaluate the contribution of the secretory pathway and the cytoskeleton for TuMV intercellular movement.

Using our dual gene cassette construct (Fig. 1), we first observed the green fluorescence at 48 hpinf, and mCherry fluorescence at 60 hpinf. This is the time frame normally observed when virus infection is initiated through agroinfiltration [64]–[66]. TuMV intercellular movement was observed between 60 and 72 hpinf and progressed at a rate of one new infected cell per ∼3 hours and systemic infection of the plant occurred at 4–5 dpinf. The intercellular rate of spread of TuMV was very close to that observed for TMV and TEV [5], [34]. Infections caused by mechanical inoculation with virus suspensions as opposed to agroinfiltration often result in measurable virus replication at 24 hpinf and systemic infection 2 days later [34]. The delay in observable infection in our agroinfiltration system may be explained by several factors. The first factor is that a T-DNA copy of the viral RNA genome is delivered in the cell. This T-DNA molecule must be transported to the nucleus and transcribed into RNA, which is then transported back in the cytoplasm. In the case of potyviruses, there may be an additional delay because the RNA transcribed from the T-DNA is not linked to VPg. There is consequently a first round of translation that needs to take place before *bona fide* infection begins. Lastly, monitoring fluorescence as opposed to viral RNA through an amplification system (e.g. RT-PCR) is likely less sensitive and requires maturation of the fluorescent marker [67]. Importantly, however, after the initial delay in infection, TuMV intercellular movement and systemic infection proceeded at the same rate as infections using purified viral particles. This indicates that the infiltrated agrobacterium did not cause an additional defense response by the plant that significantly impeded spread of TuMV beyond what is normally observed during virus infections. This latter finding further supports the use of our dual cassette construct as a valid tool to study virus intercellular movement.

It was previously shown that intracellular motility of individual potyviral proteins was dependent on the early secretory pathway [17], [39], [41], [57]. In addition to ER, COPI, and COPII coatomers, the Golgi apparatus can be recruited into virus factories [16], [48], [68] but the role of late secretory pathway in plant viral movement was not investigated. Although ESCRT (endosomal sorting complexes required for transport) proteins, which have a major role in the sorting of cargo proteins, are recruited for tomato bushy stunt virus replication [69], [70], it is not known if they have any involvement in virus movement. In the present study, we determined that in addition to the early secretory pathway, post-Golgi transport components were also required for TuMV intercellular movement (Fig. 2–3). Likely, this late secretory pathway is required for sorting the membrane-associated viral RNA-protein complex to PDs.

Intercellular movement of TuMV also depended on microfilaments (Fig. 6) and myosin motor proteins (Fig. 7 and 8). In plant cells, myosins are classified into class VIII or class XI [71]. Among the myosins tested in this study, myosin XI-2 and XI-K were required for TuMV intercellular movement, but not myosin XI-F, VIII-1 or VIII-2 [39]. Class XI myosins are also required for normal sustained movement of TMV [22] and GFLV [6]. In the case of GFLV, it may be that myosin is required to transport a host factor to the PD that then supports GFLV movement. For TMV, it has been suggested that the influence of myosin XI-2 on its sustained intercellular spread may be through metabolism of virus products after virus movement, since the related tobamovirus TVCV does not require actomyosin for intercellular spread and TMV spreads normally for 24 h post treatment with a microfilament antagonist [22], [61], [72]. Previously, myosin XI-K was shown to be involved in the intracellular movement of TuMV 6K_2_ vesicles [41]. Myosin XI-K and myosin XI-2, but not other myosins, have been shown to be major facilitators for cellular motility between actin filaments and the ER [73]. It has also been shown that myosin XI-K localizes to the motile endomembrane vesicles associated with F-actin [71]. We suggest that there may be more than one myosin-dependent activity necessary for a single virus and its expressed proteins to spread in plants and that XI-2, together with XI-K, may be important for the movement of TuMV RNA complexes.

Disruption of the secretory pathway had no impact on TuMV accumulation in the initially infected cells. We showed previously that BFA treatment had no effect on the formation of TuMV-induced 6K_2_-tagged structures, although motile 6K_2_ vesicles showed a higher incidence of localization with the COPII marker Sec24 [48]. Similarly, disruption of the early secretory trafficking by BFA inhibited intercellular virus movement of melon necrotic spot virus but did not modify its accumulation in infected cells [16]. Coronavirus-induced remodeling of the ER and viral replication equally took place in the presence of BFA [74]. Breakdown of actin filaments also did not affect the formation of TuMV 6K_2_-tagged vesicles [40]. These results suggest that replication activities, despite their requirement for membranes, are influenced separately from those involved in movement, although aspects of both are likely coordinated [75].

In conclusion, we show in this study that the secretory pathway and the actomyosin system are both important for the intercellular movement of TuMV. These host components are likely required by the virus to aid its movement out of the initially infected cell. Further work is necessary to identify host proteins within the secretory pathway and the actomyosin network that interact with the virus proteins and influence virus movement.

## Materials and Methods

### Fluorescent proteins and molecular clones

TuMV infectious clone pCambiaTuMV/6K_2_:mCherry was as described [40], [76]. Ara7/RabF2b was as described [59]. The binary vectors designed to express *N. benthamian*a myosin tails VIII-1, XI-K, XI-F, and XI-2 were as described [23]. The introduction of the 35S-GFP-HDEL gene cassette into pCambiaTuMV/6K_2_:mCherry was done as follows: pBIN/20-ER-gk [77] was digested with *Ase*I and ligated with similarly digested pCambiaTuMV/6K_2_:mCherry. Kanamycin-resistant *Escherichia coli* colonies were screened for pCambiaTuMV/6K_2_:mCherry//GFP-HDEL.

### Protein expression in plants

Transient expression studies were performed by agroinfiltration on three-week-old *N. benthamiana* plants. Plasmids were introduced by electroporation into *Agrobacterium tumefaciens* AGL1 and selected on LB ampicillin-kanamycin plates. The pellet of an overnight culture was gently suspended in water supplemented with 10 mM MgCl_2_ and 150 µM acetosyringone and left at room temperature for 3 h. The solution was then diluted to an OD_600_ of 0.6 for pCambiaTuMV/6K2:mCherry//GFP-HDEL; 0.1 for pARF1(NI), pRAB-E1d(NI) and pYFP-RAB-F2^b^; 1.5 for pTRV1 and pTRV2; 0.3–0.5 for myosin dominant negative mutant. For co-expression, 1∶1 mixture of the two AGL1 bacteria containing the plasmids of interest were agroinfiltrated. All dominant negative mutants were agroinfiltrated 24 h before pCambiaTuMV/6K_2_:mCherry//GFP-HDEL agroinfiltration. Plants were kept for 3–4 dpinf in a growth chamber until observation.

### FM4-64 staining

Small pieces of *N. benthamiana* leaves were cut and dipped in 1 µg/µl of FM4-64 (Molecular Probes). Leaves were incubated at room temperature for 40–45 minutes and observed by confocal laser microscopy.

### Inhibitor treatment

Stock solutions of Latrunculin B (LatB: 2.5 mM Calbiochem) and Cytochalasin D (CytD; 20 mM Calbiochem) were prepared in dimethyl sulfoxide (DMSO) and diluted to the desired concentration in water prior to their infiltration into 3-week-old *N. benthamiana* leaves. The final concentration of Brefeldin A (BFA), CMA, Tyrphostin A23, Tyrphostin A51 and Wortmannin were 10 µg/ml, 0.5 µM, 30 µM, 30 µM, and 20 µM, respectively. The maximal surface of N. benthamiana leaves were agroinfiltrated with the inhibitors 4 hours before pCambiaTuMV/6K_2_:mCherry//GFP-HDEL agroinfiltration. pCambiaTuMV/6K_2_:mCherry//GFP-HDEL agroinfiltration was restricted to a small region in the leaf, to be sure that this region received the inhibitors treatment, and in order to be able to follow the cell to cell movement.

### VIGS and quantitative RT-PCR

pTRV2 with myosin fragments was as described [22]. Virus-induced gene silencing (VIGS) studies were conducted as described previously [78], [79]. vTRV infections were established in *N. benthamiana* by co-agroinfiltration of pTRV1 and pTRV2. To confirm silencing of specific myosin transcripts, RNA was isolated from 20 day-old TRV-infected systemic leaves (two plants/construct) using the RNeasy plant mini kit (Qiagen). DNase-treated RNA (4 µg) was used to generate cDNA with iScript cDNA synthesis kit (Bio-Rad). After a 15-fold dilution of the cDNA, 2 µL of solution was used for quantitative RT-PCR through a Rotor Gene 3000 real-time DNA detection system (Corbett Research). The following primers were used to detect *N. benthamiana* myosins: VIII-1: 5′-GCCCGAGAGAGCAATGGA-3′and 5′-CCTCAGCTAATCGGCTTATAACACT-3′; VIII-2: 5′-ACTCCTATTGAATTTGCCAGCAA-3′ and 5′-CTGCACATAAACTGCCATTATTCC-3′; XI-2: 5′-CAACTCCTACCCGCAAACCA-3′ and 5′-TCCCATTGTCATTCTCCCAAA-3′; XI-F: 5′-GCACAGGGTTTTCGCTCAA-3′ and 5′-CCCTCAATTCCGCTGTATCC-3′. Transcript levels were adjusted for loading differences after comparison with Actin-Binding Domain 2(ABD2) transcript internal control values and were calculated using the Delta-Delta CT method. *N. benthamiana* leaves above the original TRV-inoculated leaf were agroinfiltrated with pCambiaTuMV/6K_2_mCherry//GFP-HDEL 16 days after TRV infection. The leaves were observed 4 days later by confocal microscopy.

### Confocal microscopy

Agroinfiltrated leaf sections were mounted on a depression microscope slide, aligning the leaf tissue in the well. Cells were observed using a 10× objective, 20×, 40× and 63× oil immersion objective on a LSM 510 Metaconfocal microscope (Zeiss) or on a LSM 780 Metaconfocal microscope (Zeiss). For LSM 510 Metaconfocal microscope experiments, argon and HeNe lasers were used to excite fluorescent proteins and for a LSM 780 Metaconfocal multiline argon and DPSS 561 were used. Data from both green and red channels were collected at the same time.

After acquisition, images were processed using Metamorph and/or ImageJ to quantify the average intensity of fluorescence, Carl Zeiss LSM Image Browser, and/or Adobe Photoshop software for post-capture imaging processes.

### Statistical analyses

Statistical analysis was performed from a total of 10–21 patches from 21 leaves and 5 different plants. Graphpad Prism One-way analysis of variance (1 way ANOVA) was used to assess the overall statistical differences between the means of different groups. Following 1 way ANOVA, Tukey's Multiple Comparison Test was also used to assess whether the mean of two particular groups were different from each other. P value summary (P<0.05) shows statistically significant differences between different treatments.

## Supporting Information

Figure S1 (A) The TuMV cassette in pCambiaTuMV/6K_2_:mCherry//GFP-HDEL was replaced by 6K_2_:mCherry cassette. Single-slice confocal microscope images of *N. benthamiana* leaf agroinfiltrated 4 days before with *A. tumefaciens* strain Agl1 containing the above plasmid. Left panel, red fluorescence channel imaging 6K_2_:mCherry; middle panel, green fluorescence channel imaging GFP-HDEL; and right panel, merged images. Scale bar = 200 µm. (B) Three-dimensional rendering of 30 1 µm thick confocal images that overlap by 0.5 µm of *N. benthamiana* agroinfiltrated leaves showing distribution of ERD2 in Golgi bodies (left panel) and its retention in ER following BFA treatment. Scale bar = 20 µm. (C) Three-dimensional rendering of 30 1 µm thick confocal images that overlap by 0.5 µm of *N. benthamiana* agroinfiltrated leaves showing FM4-64-labeled endocytic vesicles (arrows, left panel) and inhibition of FM4-64-labeled endocytic vesicle formation by 20 µM Worthmannin treatment 4 h prior to staining (right panel). Scale bar = 20 µm. (D) Three-dimensional rendering of 30 1 µm thick confocal images that overlap by 0.5 µm of *N. benthamiana* agroinfiltrated leaves showing distribution of actin microfilaments in the presence of DMSO (left panel), 10 µM CytD (middle panel) and 5 µM LatB (right panel). Scale bar = 20 µm.
(TIF)

Figure S2 (A–C) Repeated experiment as described for Fig. 2D, Fig. 3D and Fig. 4E, respectively. One-way analysis of variance calculation followed by Tukey's Multiple Comparison Test allowed analysis of differences between means: = NS, not significant, ***, 0.0001<P value<0.001, **, 0.001<P value<0.01, *, P<0.05.
(TIF)

Figure S3 (A–C) Repeated experiment as described for Fig. 6D, Fig. 7B and Fig. 8A, respectively. One-way analysis of variance calculation followed by Tukey's Multiple Comparison Test allowed analysis of differences between means: = NS, not significant, ***, 0.0001<P value<0.001, **, 0.001<P value<0.01, *, P<0.05.
(TIF)

Movie S1 Time lapse series of three-dimensional rendering tile confocal image of *N. benthamiana* leaf agroinfiltrated 72 hrs before with *A. tumefaciens* strain Agl1 containing pCambiaTuMV/6K_2_:mCherry//GFP-HDEL. The confocal image tiles was formed using the 10× objective by assembly 5×5 images in xy and the three-dimensional rendering was created by 5 z stacks of 90 µm thick confocal images that overlap by 45 µm. Images were taken every hour for 17 consecutive hours, indicated at upper left. Scale bar = 1 mm
(AVI)
