# Supplementary figures and images for: Contribution of Host Intracellular Transport Machineries to Intercellular Movement of Turnip Mosaic Virus
Source: PLoS Pathog. 2013 Oct 3;9(10):e1003683. doi: 10.1371/journal.ppat.1003683 (PMC3789768; doi:10.1371/journal.ppat.1003683)

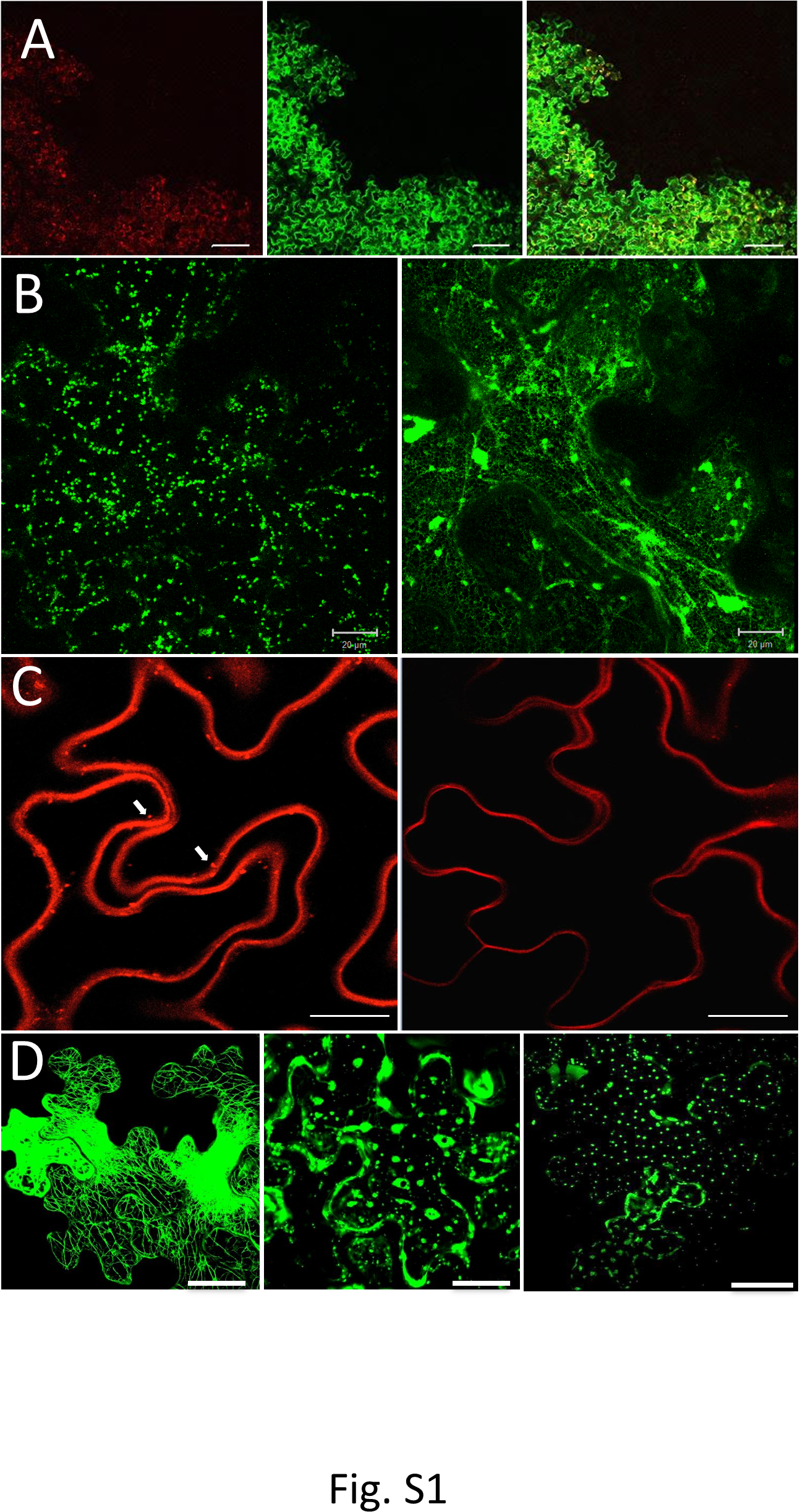

Supplement: Figure S1 — (A) The TuMV cassette in pCambiaTuMV/6K2:mCherry//GFP-HDEL was replaced by 6K2:mCherry cassette. Single-slice confocal microscope images of N. benthamiana leaf agroinfiltrated 4 days before with A. tumefaciens strain Agl1 containing the above plasmid. Left panel, red fluorescence channel imaging 6K2:mCherry; middle panel, green fluorescence channel imaging GFP-HDEL; and right panel, merged images. Scale bar = 200 µm. (B) Three-dimensional rendering of 30 1 µm thick confocal images that overlap by 0.5 µm of N. benthamiana agroinfiltrated leaves showing distribution of ERD2 in Golgi bodies (left panel) and its retention in ER following BFA treatment. Scale bar = 20 µm. (C) Three-dimensional rendering of 30 1 µm thick confocal images that overlap by 0.5 µm of N. benthamiana agroinfiltrated leaves showing FM4-64-labeled endocytic vesicles (arrows, left panel) and inhibition of FM4-64-labeled endocytic vesicle formation by 20 µM Worthmannin treatment 4 h prior to staining (right panel). Scale bar = 20 µm. (D) Three-dimensional rendering of 30 1 µm thick confocal images that overlap by 0.5 µm of N. benthamiana agroinfiltrated leaves showing distribution of actin microfilaments in the presence of DMSO (left panel), 10 µM CytD (middle panel) and 5 µM LatB (right panel). Scale bar = 20 µm. (TIF) [file ppat.1003683.s001.tif]

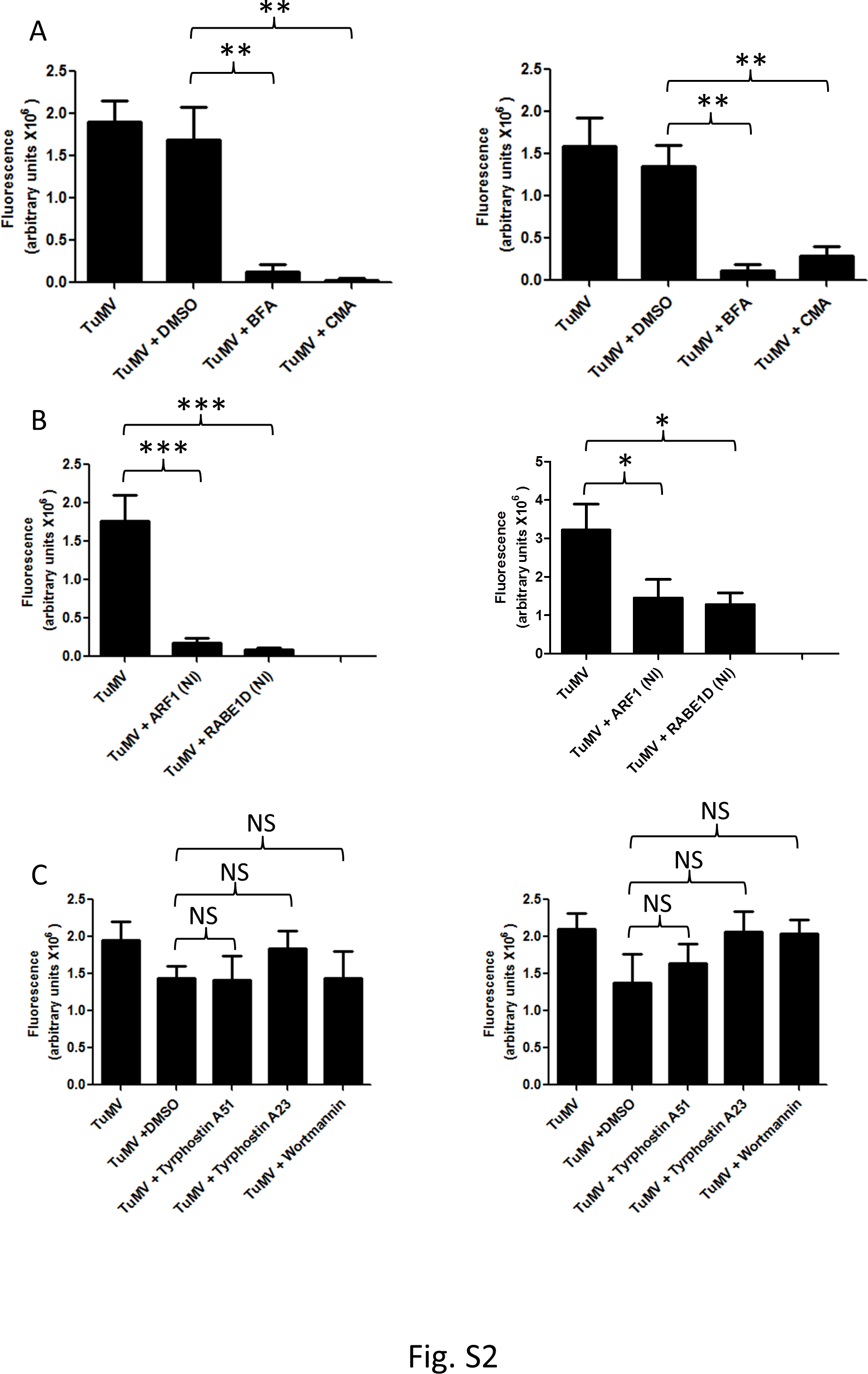

Supplement: Figure S2 — (A–C) Repeated experiment as described for Fig. 2D, Fig. 3D and Fig. 4E, respectively. One-way analysis of variance calculation followed by Tukey's Multiple Comparison Test allowed analysis of differences between means: = NS, not significant, ***, 0.0001<P value<0.001, **, 0.001<P value<0.01, *, P<0.05. (TIF) [file ppat.1003683.s002.tif]

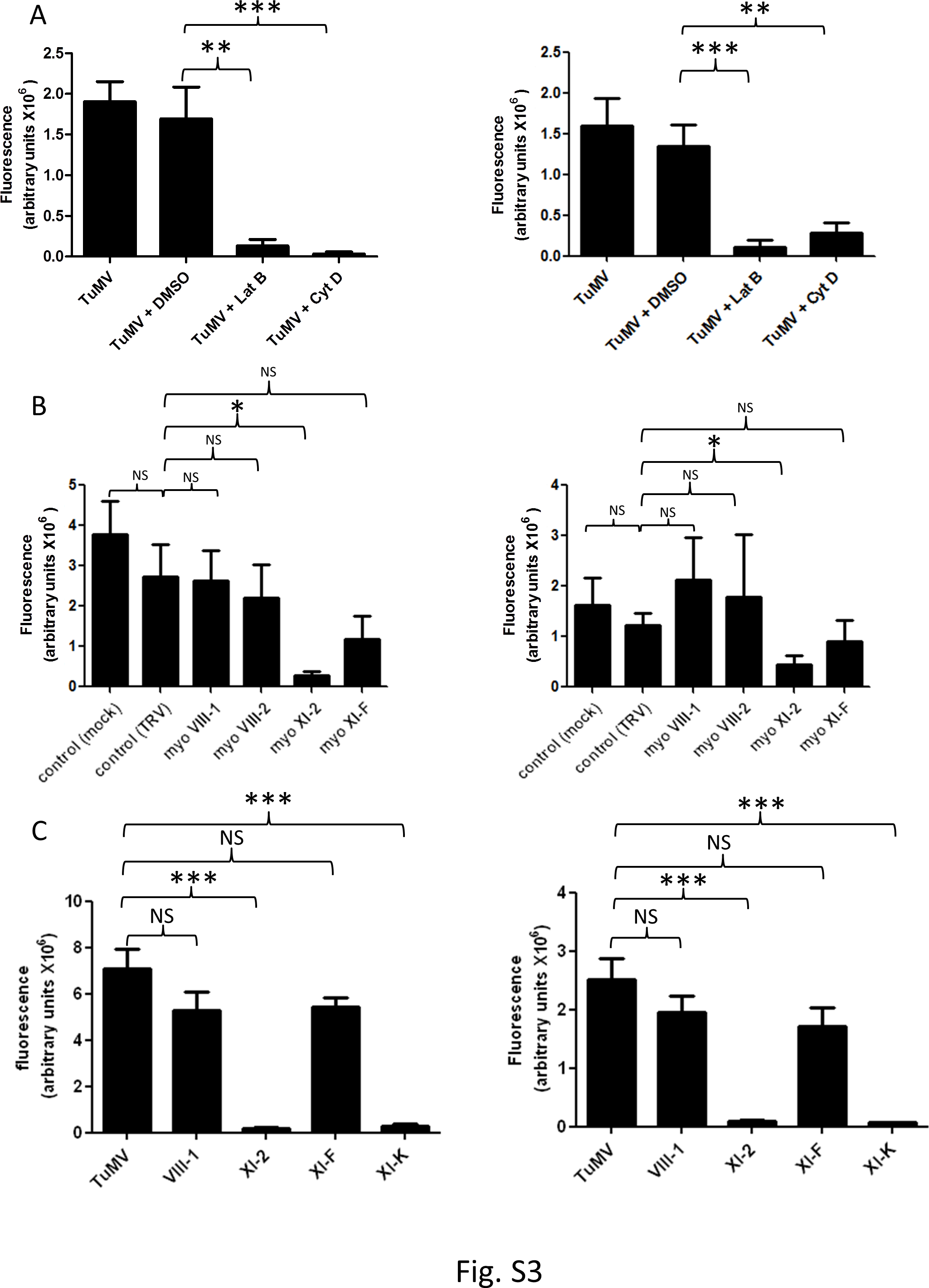

Supplement: Figure S3 — (A–C) Repeated experiment as described for Fig. 6D, Fig. 7B and Fig. 8A, respectively. One-way analysis of variance calculation followed by Tukey's Multiple Comparison Test allowed analysis of differences between means: = NS, not significant, ***, 0.0001<P value<0.001, **, 0.001<P value<0.01, *, P<0.05. (TIF) [file ppat.1003683.s003.tif]
